# Highly Multiplexed Digital Spatial Profiling of the Tumor Microenvironment of Head and Neck Squamous Cell Carcinoma Patients

**DOI:** 10.3389/fonc.2020.607349

**Published:** 2021-01-19

**Authors:** Arutha Kulasinghe, Touraj Taheri, Ken O’Byrne, Brett G. M. Hughes, Liz Kenny, Chamindie Punyadeera

**Affiliations:** ^1^ The School of Biomedical Sciences, Institute of Health and Biomedical Innovation, Queensland University of Technology, Kelvin Grove, QLD, Australia; ^2^ Translational Research Institute, Brisbane, QLD, Australia; ^3^ Department of Pathology, Royal Brisbane and Women’s Hospital, Brisbane, QLD, Australia; ^4^ Royal Brisbane and Women’s Hospital, Herston, QLD, Australia; ^5^ Princess Alexandra Hospital, Woolloongabba, QLD, Australia; ^6^ School of Medicine, University of Queensland, Brisbane, QLD, Australia

**Keywords:** head and neck cancer, head and neck squamous cell carcinoma, tumor microenvironment, digital spatial profiling, immunotherapy

## Abstract

**Background:**

Immune checkpoint inhibitors (ICI) have shown durable and long-term benefits in a subset of head and neck squamous cell carcinoma (HNSCC) patients. To identify patient-responders from non-responders, biomarkers are needed which are predictive of outcome to ICI therapy. Cues in the tumor microenvironment (TME) have been informative in understanding the tumor-immune contexture.

**Methods:**

In this preliminary study, the NanoString GeoMx™ Digital Spatial Profiling (DSP) technology was used to determine the immune marker and compartment specific measurements in a cohort of HNSCC tumors from patients receiving ICI therapy.

**Results:**

Our data revealed that markers involved with immune cell infiltration (CD8 T-cells) were not predictive of outcome to ICI therapy. Rather, a number of immune cell types and protein markers (CD4, CD68, CD45, CD44, CD66b) were found to correlate with progressive disease. Cross platform comparison with the Opal Vectra (Perkin Elmer) for a number of markers across similar regions of interest demonstrated concordance for pan-cytokeratin, CD8, and PD-L1.

**Conclusion:**

This study, to our knowledge, represents the first digital spatial analysis of HNSCC tumors. A larger cohort of HNSCC will be required to orthogonally validate the findings.

## Introduction

Head and neck cancers account for 700,000 new cases a year, resulting in 380,000 deaths worldwide. Recently, recurrent or metastatic squamous cell carcinomas of the head and neck (HNSCC) had FDA approval for anti PD-1 immune checkpoint inhibitors (ICI) Nivolumab and Pembrolizumab for patients that were refractory to platinum agents. In 2019, approval was also granted for Pembrolizumab as a first line treatment for patients with metastatic or unresectable, recurrent HNSCC whose tumors expressed PD-L1 combined positive score ≥1 (1–4). ICI treatment is cost intensive and associated with potential immune-related adverse effects, therefore identifying patients likely to respond is paramount. There remains a need for biomarkers which are able to select the most suitable patient for ICI therapy in HNSCC (5–8).

A number of factors dictate how well a HNSCC tumor may respond to ICI therapy: (i) the immune contexture of the tumor microenvironment (TME) which includes the type, density, location, phenotypic, and functional profile of immune cells (9–11) and; (ii) the extent of mutations in the tumor cells (tumor mutation burden – TMB) (12–14). Studies have demonstrated that the immune contexture and spatial profiles can impact directly on the clinical response to ICI therapy. When a tumor has a high degree of T-cell infiltration, it is generally termed a “hot tumor”, where the immune system has recognised the tumor cells which in turn may be easier to target and treat with ICI. In comparison, “cold”, or cold-acting tumors tend to be harder to treat as they have fewer mutations and checkpoint proteins that do not engage with the immune system to attack the cancer cells (15). Numerous studies have shown that tumor PD-1/PD-L1 status varies significantly and is often non-predictive (1). In addition, PD-L1 expression and TMB have non-overlapping effects on the response to PD-1/PD-L1 inhibitors and were found to be independent of TMB (16). These studies highlight how each biomarker may point to differing mechanisms informative of response to ICI therapy (17).

Interrogation of the tumor microenvironment has become a powerful tool in understanding cellular interactions which are key for ICI to be effective in tumor types such as HNSCC, non-small cell lung cancer and melanoma. Recently, multiplex immunofluorescence (mIF) has come to the fore to spatially discern the tumor microenvironment from formalin-fixed paraffin-embedded (FFPE) tumor tissue. To this end, digital spatial profiling has been developed by Nanostring Technologies which uses DNA oligo tags that are covalently linked to primary antibodies *via* a UV photocleavable linked to identify targets *in situ* and enable quantitation *via* nCounter technology *ex situ*. For this proof-of-principle study in HNSCC, we analyzed a number of HNSCC patient tumors using the NanoString GeoMx DSP technology using the Immuno-oncology profiling core which covers protein targets across Immuno-oncology (IO) drug targets, immune activation status, immune cell typing, and pan-tumor modules. Our data shows that multidimensional spatial analysis of HNSCC tumors reveals a number of cell types (CD4, CD68, CD45, CD44, CD66b) which were found to associate with progressive disease to ICI therapy.

## Materials and Methods

### Patient Recruitment

This study was conducted at the Royal Brisbane and Women’s Hospital (RBWH) in Queensland, Australia. Ethical approval was obtained from the Metro South Health District Human Research Ethics Committee in accordance with the National Health and Medical Research Councils guidelines (Ethics Approval HREC/12/QPAH/381) and a site specific agreement with RBWH. The study has institutional approval from the Human Ethics Committee (1400000617). Following written informed consent, tumor tissue (FFPE sections) were collected from n=7 HNC patients receiving ICI therapy (Nivolumab or Pembrolizumab) for metastatic disease. The clinical outcomes following ICI therapy was reported as non-progressors (NP) or progressive disease (PD).

### Tissue Preparation

FFPE tissue (8 unstained x 5 µm) were prepared onto charged slides by Queensland pathology and one slide was H&E stained. The H&E slide was scanned at 20x and 40x magnification on the 3D Histech slide scanner and sent for demarcation of tumor and normal regions by a qualified pathologist. Four unstained slides were used for the Opal Multiplex IF staining (PerkinElmer) and two unstained slides were transported to Nanostring Technologies (Seattle) for DSP profiling.

### Multispectral Immunofluorescence

Multispectral immunofluorescence staining was performed using the Opal four-color IHC Kit (NEL794001KT; Perkin Elmer, Waltham, MA) as per manufacturer’s instructions. The Opal kit uses tyramide signal amplification (TSA) conjugated fluorophores to detect targets within an immunofluorescence assay. Briefly, the slides were deparaffinised with Xylene, rehydrated with a series of graded ethanol (100%, 95%, 70% for 2 min) and washed in Tris buffered saline with 0.1% Tween 20 (Merck). Slides were then heated using microwave treatment (MWT) for 45 seconds at 100% power followed by 15 min at 20% power in AR Buffer. Slides were blocked using the blocking diluent (Perkin Elmer) for 10 min at room temperature (RT). The primary antibody was incubated for 37°C for 32 min in the Dako Slide Hybridiser (Agilent, Ca, USA). The primary antibodies used in the study were from Cell Signalling Technology: pan keratin (C11, cat #4545) Mouse mAb (1:750 dilution), CD8 (C8/144B, cat #70306) Mouse mAb (1:1,000 dilution), PD-L1 (E1L3N XP, cat #13684) Rabbit mAb (1:500 dilution). Slides were then washed in TBST with agitation and incubated with Opal Polymer HRP MS+Rb (Perkin Elmer) for 10 min at RT. Opal signal was generated by incubating the slides in the Opal working solution (Opal 520, Opal 570, Opal 670; 1:50 dilution) for 10 min. Slides were washed in TBST and underwent MWT in AR buffer prior to probing with the next antibody. Slides were then counterstained with DAPI for 5 min and mounted with Prolong Gold (Invitrogen) and coverslipped prior to imaging. Slides were imaged using the Vectra 3.0 spectral imaging system (PerkinElmer) and image analysis performed using the InForm image analysis software (PerkinElmer). Low magnification scanning at 10x was initially performed to get an overview of the slide and to compare to pathologist-demarcated H&E sections, next 10 regions of interest across the fields were chosen across tumor, immune, infiltrating edges, and the stroma to get representative images of the tissue section.

### Protein Digital Spatial Profiling

**Figure 1:**
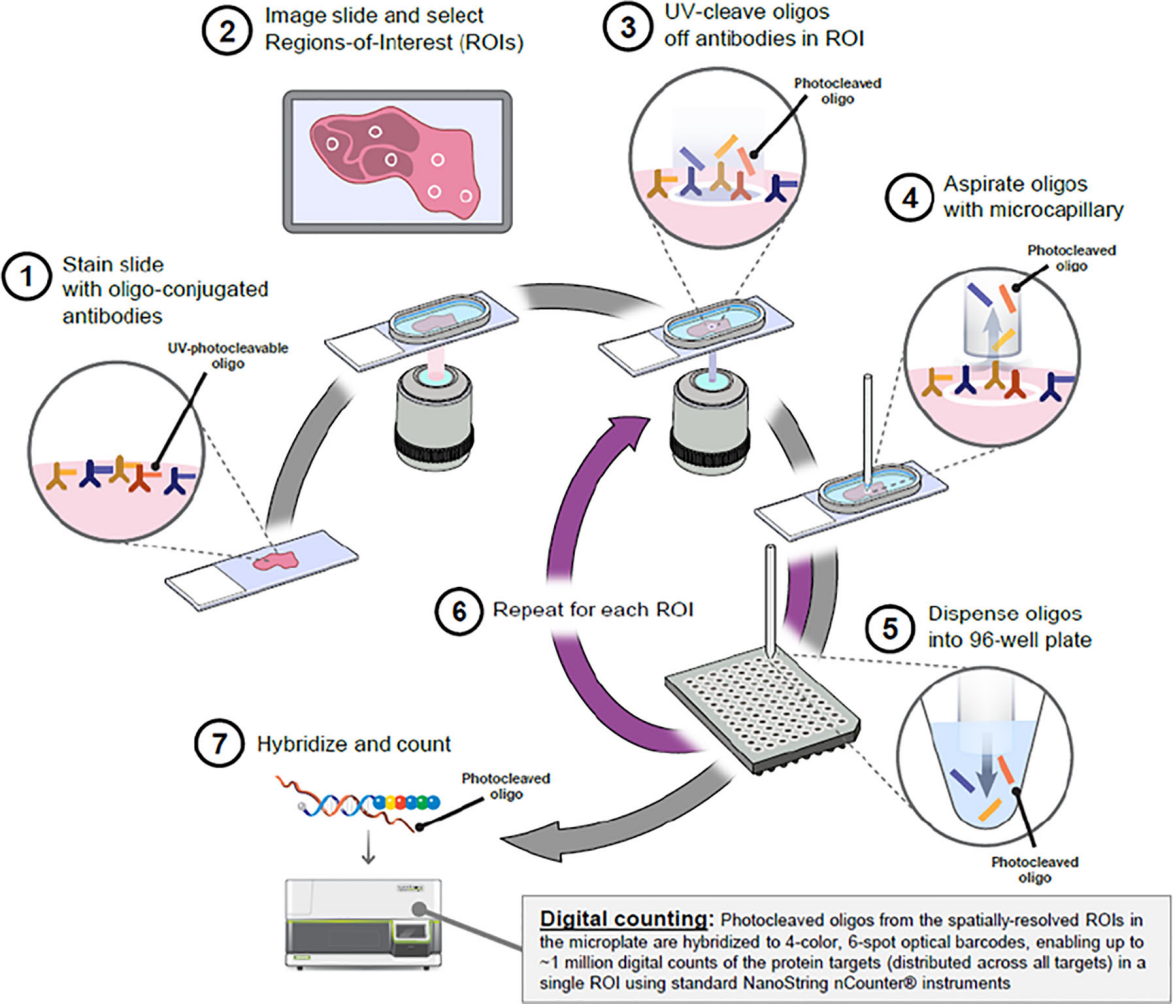
Overview of the Digital Spatial Profiling (DSP) platform (Nanostring Technologies, Seattle). Antibodies are covalently bonded to a DNA indexing oligo with a UV photo-cleavable linker. These reagents are used to stain HNSCC tumor tissue sections and focused UV light can liberate the indexing oligos from the regions of interest (ROI). The oligos can be collected and digitally counted. Image obtained with permission from Merritt et al., bioRxiv 2019.

The DSP workflow was carried out by Nanostring Technologies as per Merritt et al., BioRxiv 2019 (**Figure 1**). In brief, the protocol has five steps: standard FFPE tissue preparation; tissue incubation with a mixture of visualization markers (VMs) and DSP probes; imaging and region of interest (ROI) selection; ultraviolet (UV) exposure; and oligo collection step. This is then quantified using the Nanostring nCounter^®^ system. Once the VM step have been completed to visualize the tissue morphology based on four-color fluorescence imaging of epithelial cells (PanCK), T cell (CD3), cytotoxic T cells (CD8), and nuclear stain (DAPI), twelve regions of interest (ROI) at 200µm circular diameter were selected in consultation with a pathologist. After UV illumination of the ROIs, the eluent was collected *via* microcapillary aspiration and transferred into individual wells of a microtiter plate. Once the 12 ROIs were processed, indexing oligos were hybrisised to NanoString optical barcodes for digital counting on the nCouter^®^. Digital counts from barcodes corresponding to protein probes were then normalized to ERCC and housekeeping counts.

### Whole Exome Sequencing

tumor tissue slides were profiled using whole exome sequencing (xGEn, Cat#1506114) at the Australian Translational Genomics Centre (ATGC) with a mean depth of 61x for the exome and a xGen Pan-Cancer Panel v2.4 (Integrated DNA Technologies, US) covering 523 clinically relevant gene with a mean coverage of 202x. The tumor mutation burden (TMB) was calculated using variants in the high coverage panel including only variants generating protein coding alterations. To calculate the TMB, the total number of somatic nonsynonymous mutations was normalized to the total number of megabases sequenced.

### Statistical Analysis

Statistical anaylsis was performed using GraphPad Prism (version 8.2.1). All statistical tests used a cut-off P-value of 0.05 for significance and were two sided. Unpaired t-test was used to compare the ROI findings to the clinical outcome data.

## Results

### Patient Characteristics

The patient clinicopathological findings are presented in **Table 1** (staged according to the 8^th^ TMN edition). FFPE slides were collected from n=7 metastatic HNSCC patients prior to commencement of therapy. The HNSCC anatomical features were three oral cavity cancer (OC) and four oropharyngeal cancers (OPC) (three human papillomavirus HPV positive). Patients were treated with a combination of surgery, radio, and chemotherapy prior to ICI treatment (Nivolumab/Pembrolizumab) in the metastatic setting. The treatment response data following ICI treatment was stable disease in two patients, progressive disease in three patients and death in two patients.

**Table 1:** HNSCC clinicopathological features.

*Variables*	*N*
*Total*	7 (100%)
***Gender***	
*Male*	6
*Female*	1
***Age, y***	
*<60*	2
*>60*	5
***Anatomic site of primary tumor***	
*Oral Cavity*	3
*Oropharyngeal*	4
***Staging at diagnosis***	
*II*	1
*III*	4
*IV*	2
***HPV status***	
*HPV positive*	3
*HPV negative*	4
***Immune checkpoint therapy***	
*Nivolumab*	2
*Pembrolizumab*	5
***Patient Outcome***	
*Stable disease*	2
*Progressive disease*	3
*Deceased*	2

### Multiplex-IF Characterization (Opal PerkinElmer and Nanostring GeoMX™ DSP)

**Figure 2:**
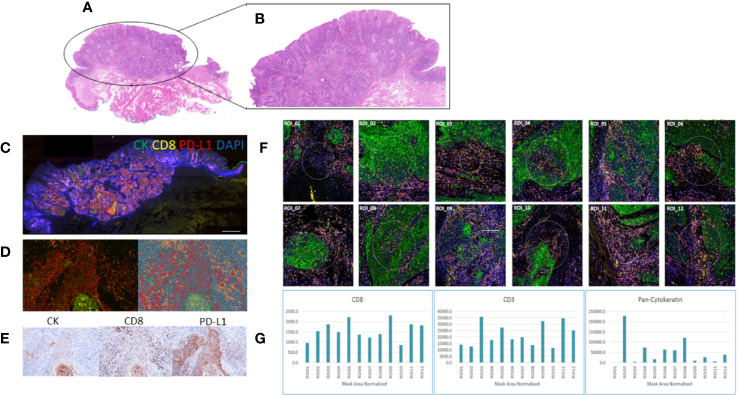
Comparative staining across two multiplex technologies. **(A)** H&E stain of head and neck squamous cell carcinoma (HNSCC) tumor tissue; **(B)** highlighting the tumor region; **(C)** Opal four-color IHC staining (PerkinElmer) of the HNSCC tissue section for cytokeratin (green), CD8 (yellow), PD-L1 (red), DAPI (blue); **(D)** a tumor ROI imaged at 20x; **(E)** indicative brown stains of the individual channels for CK, CD8, and PD-L1; **(F)** whole slide imaged with visualization markers for ROI selection on the Nanostring GeoMx DSP 12 regions of interest (ROI) selection in multiple tumor regions; **(G)** barcode counts per ROI across the visualization markers CD8, CD3, and pan-cytokeratin.

Four color tumor tissue profiling and whole slide scanning was performed on a subset of serial sections of HNSCC tissue to compare the protein expression profiles between the Opal PerkinElmer kit (Cytokeratin, CD8, PD-L1, and DAPI) and Nanostring GeoMX DSP (Cytokeratin, CD8, CD3, and DAPI) (**Figure 2**). Similar staining patterns were found between the cytokeratin and CD8 markers across both platforms, consistent with the tumor/normal demarcation by the pathologist. While the exact/matched ROIs could not be assessed using the two technologies, upon ROI selection and analysis, similar marker expressions were found for cytokeratin, CD8, and PD-L1.

### Multiplex-IHC Characterization (Nanostring GeoMx™ DSP)

**Figure 3:**
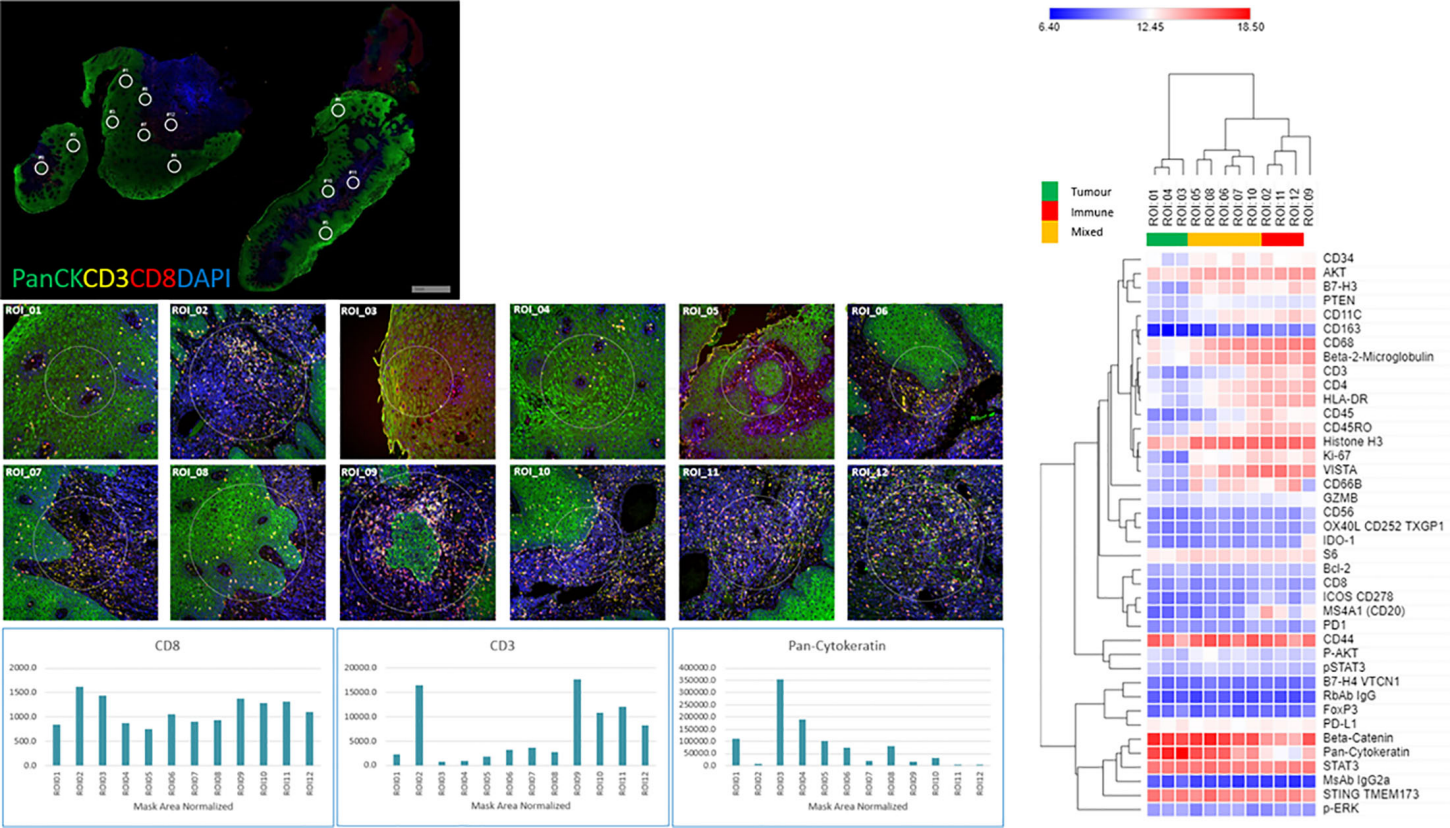
Tissue morphology (NanoString GeoMX DSP) for HNSCC tumors with 12 regions of interests (ROIs) per section, visualization markers and heatmap per tissue section. HNSCC tissue was profiled using four-color fluorescence of CD8 (red), CD3 (yellow), PanCK (green), and DNA (blue). Twelve 200 µm diameter circular regions of interest (ROIs) were selected and further profiled with a 40-plex oligo-antibody cocktail (individual ROIs below the tumor staining and counts for CD3, CD8, and PanCK cells per ROI). Unsupervised hierarchical clustering heatmaps of region-specific (tumor – green, Immune- red, Infiltrating edge with a mixed cell population – yellow) nCounter digital counts across all protein targets.

**Figure 4:**
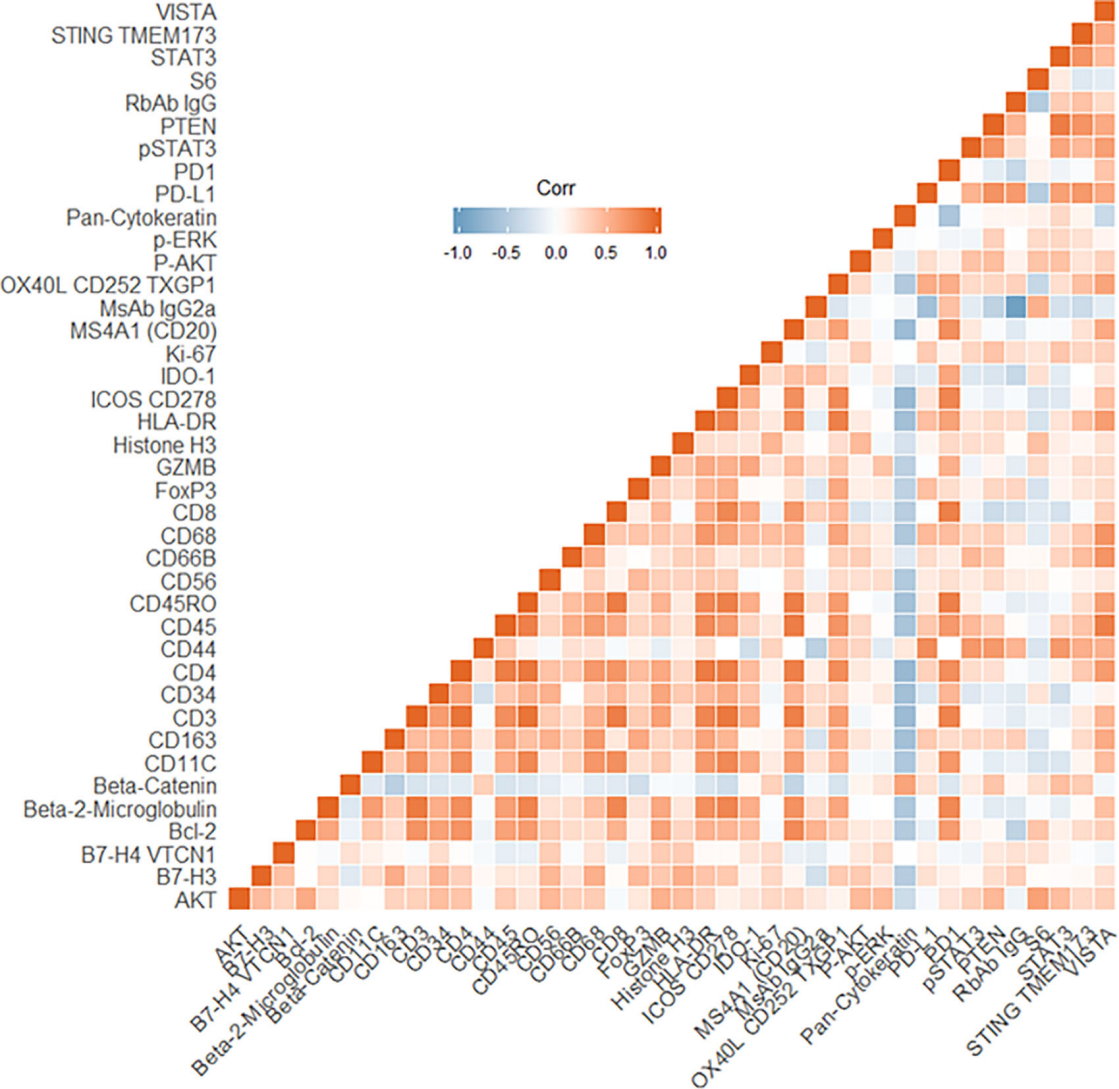
Pearson’s Correlation matrix of all target pairs analyzed across the head and neck squamous cell carcinoma (HNSCC) tumors. Red indicates a positive correlation, the blue indicates a negative correlation.

The four color tumor tissue profiling to demarcate tumor, stroma and immune regions is shown in **Figure 3**. The four color visualization markers for tumor (panCK), immune (CD3, CD8), and nuclear (DAPI) is shown per tumor section alongside the 12 ROIs. The bar graph shows the “counts” per ROI (1–12/tumor slide) for the visualization markers and these appear to have good concordance based on region selection (e.g., tumor rich areas present with high panCK counts while immune rich areas have low/no panCK counts and higher CD3/CD8 counts). Each tumor section has an unsupervised hierarchically clustered heatmap, which shows that tumor (green), immune (red), and infiltrating edge containing a mixed cell population (yellow). The tumor ROIs are enriched for pan-cytokeratin, STAT3, CD44, STING TMEM173, AKT, and CD68. The immune ROIs are enriched for CD34, BCL-2, CD45, CD11C, CD4, CD3, CD45RO, CD8, HLA-DR, B7-H3, AKT, STAT3, CD68, CD44, VISTA, and STING TMEM173. ROIs which contain mixed phenotypes such as tumor infiltrating edges are enriched for cells expressing B7-H3, CD34, CD4, CD45RO, CD3, CD8, CD68, pan-cytokeratin, AKT, CD44, HLA-DR, STAT3, and STING TMEM173. The global heatmap of the seven HNSCC tumors is shown in **Supplementary Figure 1**. Pearson’s correlation matrix of all the target pairs analyzed across the HNSCC tumors in shown in **Figure 4**. We found that pan-cytokeratin correlated strongly with PD-L1, pSTAT3, STAT3, PTEN, and STING TMEM173 and weakly with PD-1 and VISTA and immune cell types (CD8, CD68, CD4, CD3). CD8 was found to correlate strongly with PD-1, OX40L, IDO-1, ICOS CD278, HLA-DR, GZMB. Of note, the CD68, CD66B, CD45RO, and CD4 immune cell types correlated strongly with VISTA.

### Comparison of Regions of Interest

**Figure 5:**
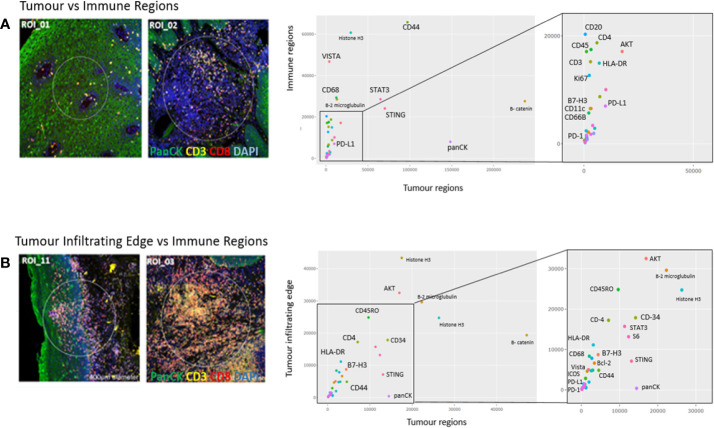
Region of interest comparison (ROI vs ROI). Tumor regions of interest plotted against **(A)** immune regions and **(B)** Tumor infiltrating edge regions. The Nanostring barcoded “oligo-counts” per marker are measured on both axes.

In **Figure 5A**, a representative comparison between a tumor ROI and immune ROI is shown, whereas in **Figure 5B**, the tumor infiltrating edge is compared to an exclusive immune cell only region in close proximity to the tumor. While the tumor regions appear enriched for pan-cytokeratin cells, the immune regions have comparatively high counts of CD68 positive cells, including increased counts for VISTA, CD44, STAT3, and STING. In the tumor infiltrating edge, an increase of AKT and CD45RO is present.

### Measurement of Markers Against Clinical Outcome

**Figure 6:**
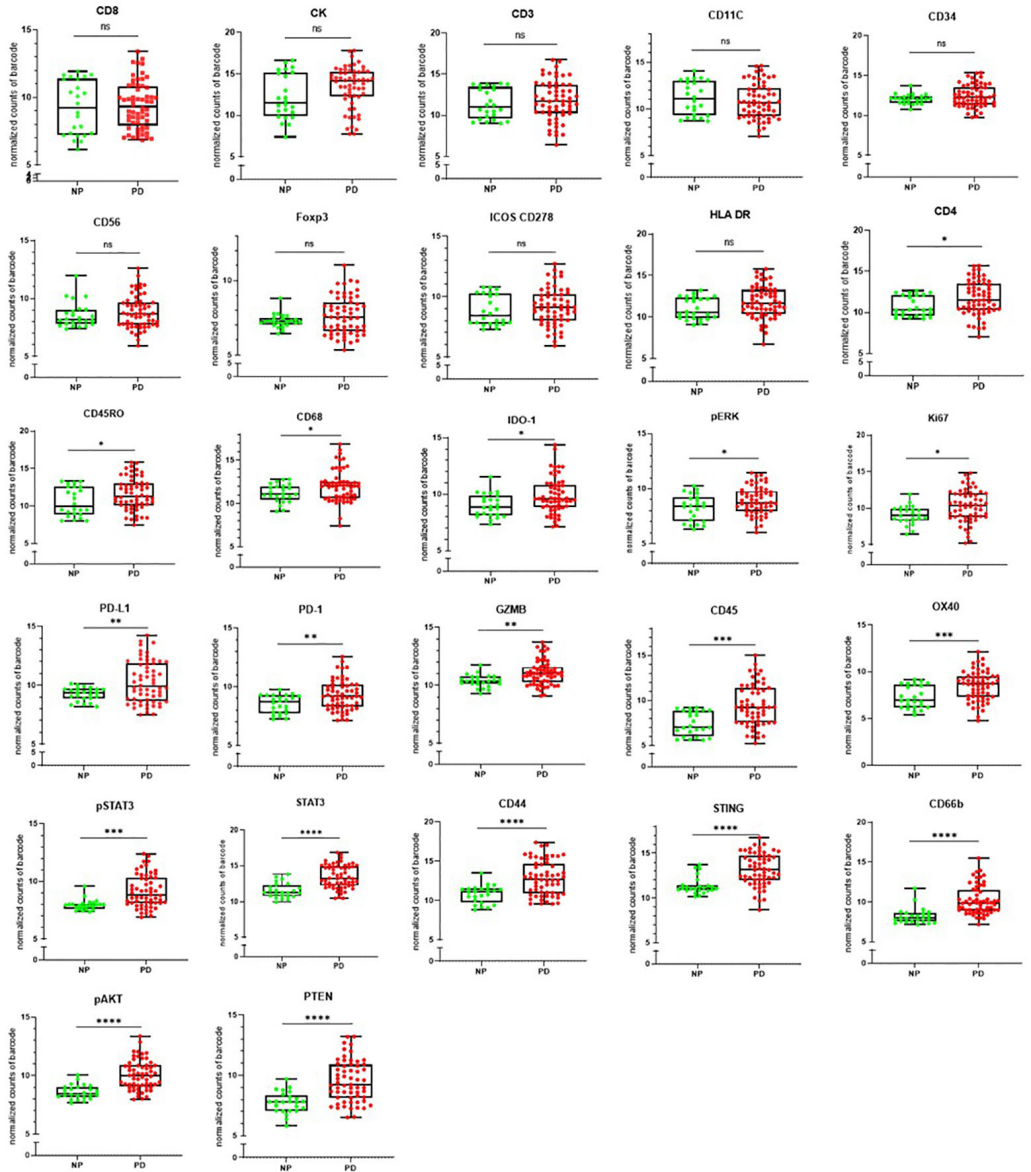
The clinical outcome following immune checkpoint therapy in the head and neck squamous cell carcinoma (HNSCC) patient cohort. [non-progressors (NP) or progressive disease (PD)] was measured per antibody target. ns=not significant p>0.05, *indicates statistical significance p ≤0.05, **p ≤ 0.01, ***p ≤ 0.001, ****p ≤ 0.0001.

The clinical outcome (NP or PD) of the patients was measured against the individual targets in the Nanostring GeoMX DSP panel as shown in **Figure 6**. CD8, pan-cytokeratin, CD3, CD11C, CD34, CD56, FOXP3, ICOS CD278, and HLA-DR were found not to be significantly associated with disease outcome. The following were found to be significantly associated with progressive disease CD4, CD45RO, CD68, IDO-1, pERK, and Ki67 (p ≤0.05); PD-L1, PD-1, GZMB (p ≤ 0.01), CD45, OX40, pSTAT3 (p ≤ 0.001); STAT3, CD44, STING, CD66b, pAKT, PTEN (p ≤ 0.0001).

### Whole Exome Sequencing of Tumors

Sufficient slides (6–8 slides/patient) were available from 3/7 HNSCC tumor samples. DNA was isolated from all three HNSCC FFPE sections for WES. Two of the three tissue sections did not progress beyond the Quality Control (QC) criteria and one sample continued to library preparation (mctc-001). The TMB of sample mctc-001 was estimated to be 5.7 mutations/Mb.

## Discussion

Identifying biomarkers predictive of response to ICI therapy has been an unmet clinical need and an area where data is emerging at a rapid pace across solid tumor biopsy and liquid biopsy in HNSCC (4, 7, 8, 18–20). Approaches using TMB and TME assessments have been used to predict outcome to ICI therapy (21). To better understand the TME, multispectral-IHC has been developed using whole slide imaging (Opal PerkinElmer and the Vectra) and higher-plex technologies such as the NanoString GeoMX DSP, which allow for 44-plex with ROI selections. There have been a few early reports using the Nanostring GeoMX DSP technology to identify markers of response to ICI therapy in melanoma (22–24), and this manuscript represents first pilot study in a small cohort of HNSCC receiving ICI.

In this preliminary study, we employed a novel digital spatial profiling methodology (NanoString GeoMX DSP) with an established multispectral IHC technique (Opal PerkinElmer) to identify predictive biomarkers of response to ICI therapy in a cohort of HNSCC patients. Across the two platforms, there was concordance between the staining patterns for a number of common markers (pan-cytokeratin, CD8, PD-L1, and DAPI) which were measured across tumor and stromal compartments. While exact ROIs were not assessable across the two methodologies, ROIs of close proximity were selected for comparative staining. The NanoString GeoMX DSP technology allows for a greater interrogation of markers compared to the Opal PerkinElmer system (44-plex vs 4-plex respectively). Therefore, to utilize the higher-plex capability of the NanoString GeoMX DSP technology, we spatially profiled seven HNSCC patient tumor biopsy FFPE samples (from patients enrolled onto ICI trials) to determine the clinical significance of a panel of 44-markers targeting immune cell profiling, immune cell activation and immuno-oncology drug target modules. The molecular compartments in the TME were defined by the detection of fluorescently labelled primary antibodies targeting pan-cytokeratin, CD8, and CD3 and DAPI.

PD-L1 was found to be highly expressed in two HNSCC tumor ROIs (mCTC-005, mCTC-006). In the same patients, across the immune cell regions, PD-1 was found to have low protein expression but higher PD-L1. Both patients received ICI (Nivolumab and Pembrolizumab) in the metastatic setting and had progressive disease at 2 years follow up. PD-L1 was found to be weakly expressed across the five other HNSCC tumor ROIs; PD-1 showed low/no expression in the tumor ROIs. Across the immune ROIs, PD-1 showed increased expression in one patient (mCTC-001) with metastatic disease to the lungs, that died 3 months after starting Pembrolizumab. This patient had low PD-L1 expression in the tumor regions. The patient was also found to have a low-intermediate TMB (5.7 muts/Mb) (13). For HNSCC, recent studies have shown that patients with a high TMB (above 10.3 muts/Mb) have associated with a higher neoantigen load on major histocompatibility complex (MHC) molecules for immune recognition and the development of an antitumor immune response (13). The comprehensive assessment of spatial PD-1/PD-L1 and TMB for this patient predicts for a poor outcome to ICI therapy. While FFPE tissue slides were collected for all patients enrolled into this study, sufficient material was available for TME assessments on all cases but limited numbers for TMB. This highlighted the importance of being able to make TME assessments from a single slide.

V-domain Ig suppressor of T cell activation (VISTA) is a novel inhibitory immune checkpoint protein that was found to strongly correlate across a number of immune cell regions in the tumor periphery of the HNSCC tumors (25) while weakly correlative within the tumor regions. VISTA functions as an immunosuppressive receptor and ligand on T-cells by decreasing IFN-γ and TNFα to block T-cell proliferation and increase the conversion of naïve to regulatory T cells. Studies have shown that combining anti-PDL1 and anti-VISTA therapies decrease tumor size and prolong survival. In addition, the study demonstrated that blockage of VISTA changes the suppressive features of the TME by decreasing the myeloid derived suppressor cells and increasing the presence of activated dendritic cells. Treatment with VISTA monoclonal antibody increased the number of tumor specific T cells in the periphery, enhanced the proliferation and infiltration into the tumor and the effector function of the tumor reactive T-cells (26).

Stimulator of interferon genes (STING) is a signaling molecule that is found in the endoplasmic reticulum, which leads to the transcription of many immune genes. STING links upstream DNA sensors to downstream IRF-3 and NF-κB pathway activation (27). It promotes an immune response against tumor cells by starting an interferon (IFN) type I over expression and is widely expressed in various cell types including T-cells, dendritic cells, and epithelial cells. In our study, STING appears to be ubiquitously expressed across tumor and immune ROIs. Emerging studies are targeting the STING pathway by utilizing STING agonists to produce IFNs to enhance the antitumor immune response (28). However, a few studies have reported on the activation of the cGAS-STING pathway which can lead to tolerogenic responses that promote tumor cell proliferation with low antigenicity through the induction of indolamine 2,3-dioxygenase (IDO) (29). In our study, IDO-1 was found to be weakly expressed across most of the tumors except for one HNSCC sample.

From the survival analysis, a number of markers were found to correlate with progressive disease (CD4, CD45RO, CD68, IDO-1, pERK, Ki67, PD-L1, PD-1, GZMB, CD45, OX40, STAT3, pSTAT3, CD44, STING, CD66b, pAKT, and PTEN). Notably, markers involved with immune cell infiltration (e.g., CD8 T-cells) were not found to be predictive of outcome to ICI therapy. However, the activity of the T-cells was not assessed at the time of sampling. While preliminary, and in a small cohort of HNSCC patients, this study provides a frame of reference for larger spatial proteomic analysis studies in HNSCC. A limitation of the study was the lack of repeat tissue biopsy to understand changes in the TME under the stressors of ICI therapy. The authors envisage a larger study of HNSCC undergoing ICI therapy where repeat tissue biopsy and metastatic tissue is available to understand the changes in the primary tissue as well as distant metastatic disease.

## Conclusion

This study demonstrates the use a novel spatial profiling methodology (NanoString GeoMX DSP) for highly-multiplexed analysis of HNSCC tumors to determine markers involved with benefit to immunotherapy. We found that the platform provided robust and reproducible data geared toward translational oncology studies where a greater depth of multi-plexing is desirable beyond conventional IHC. 

## Data Availability Statement

The datasets presented in this study can be found in online repositories. The names of the repository/repositories and accession number(s) can be found below: https://www.ncbi.nlm.nih.gov/bioproject/680183.

## Ethics Statement

The studies involving human participants were reviewed and approved by Royal Brisbane and Women’s Hospital (RBWH). Ethics Approval HREC/12/QPAH/381). The patients/participants provided their written informed consent to participate in this study.

## Author Contributions

The idea/concept was provided by AK and BH. AK, BH, LK, and TT conducted the experimentation. AK, TT, BH, and CP analyzed the data. All authors prepared and reviewed the manuscript. All authors contributed to the article and approved the submitted version.

## Funding

This study was supported by an NHMRC ECF (APP1157741) and Cure Cancer (APP1182179). The authors are grateful for the clinical trials support at the Royal Brisbane and Women’s Hospital.

## Conflict of Interest

The authors declare that the research was conducted in the absence of any commercial or financial relationships that could be construed as a potential conflict of interest.

## Supplementary Material

Supplementary Figure 1 Unsupervised hierarchically clustered global heat map of all HNSCC tumors across all ROIs.
