# Supplementary figures and images for: Highly Multiplexed Digital Spatial Profiling of the Tumor Microenvironment of Head and Neck Squamous Cell Carcinoma Patients
Source: Front Oncol. 2021 Jan 19;10:607349. doi: 10.3389/fonc.2020.607349 (PMC7851078; doi:10.3389/fonc.2020.607349)

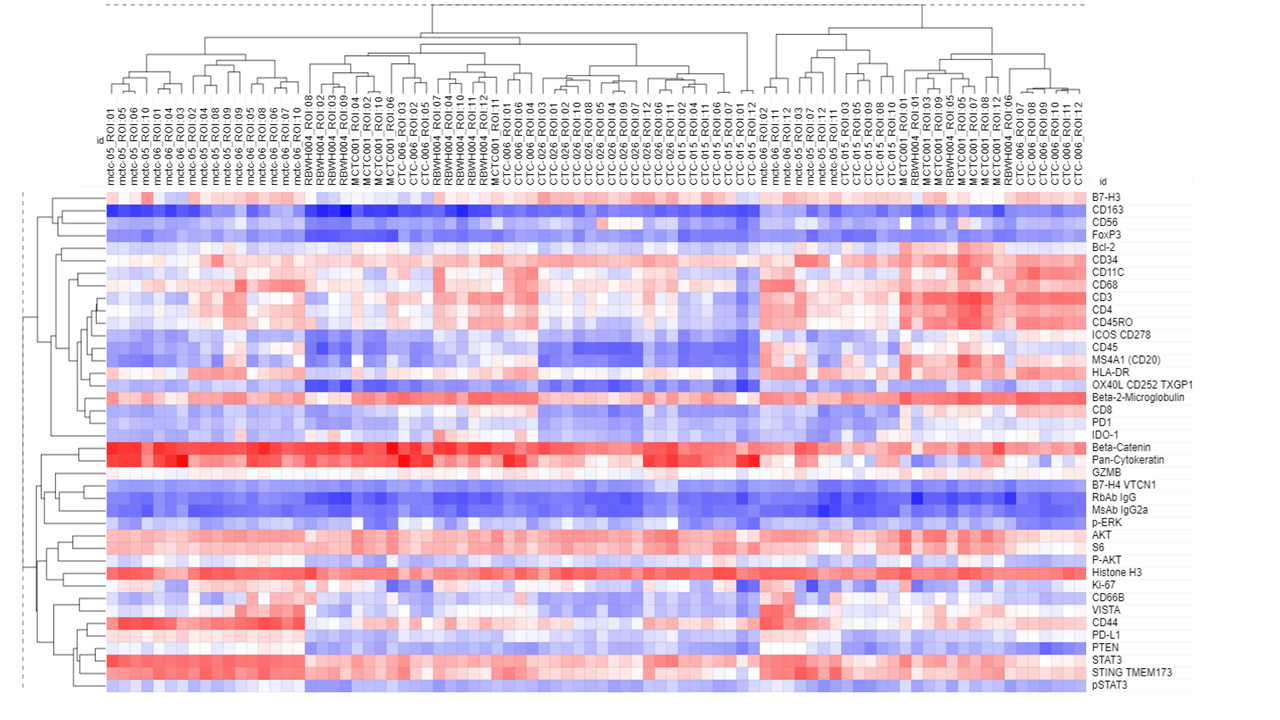

Supplement: Supplementary Figure 1 — Unsupervised hierarchically clustered global heat map of all HNSCC tumors across all ROIs. [file Image_1.tif]
